# Sinapic Acid Reduces Oxidative Stress and Pyroptosis via Inhibition of BRD4 in Alcoholic Liver Disease

**DOI:** 10.3389/fphar.2021.668708

**Published:** 2021-06-04

**Authors:** Junyi Chu, Ran Yan, Sai Wang, Guoyang Li, Xiaohui Kang, Yan Hu, Musen Lin, Wen Shan, Yan Zhao, Zhecheng Wang, Ruimin Sun, Jihong Yao, Ning Zhang

**Affiliations:** ^1^ Department of Pharmacy, The Second Hospital of Dalian Medical University, Dalian, China; ^2^ College of Pharmacy, Dalian Medical University, Dalian, China; ^3^ Department of Pharmacy, The Third Hospital of Dalian Medical University, Dalian, China

**Keywords:** alcoholic liver disease, sinapic acid, BRD4, oxidative stress, pyroptosis

## Abstract

Alcoholic liver disease (ALD) is one of the main causes of death in chronic liver disease. Oxidative stress and pyroptosis are important factors leading to ALD. Bromodomain-containing protein 4 (BRD4) is a factor that we have confirmed to regulate ALD. As a phenolic acid compound, sinapic acid (SA) has significant effects in antioxidant, anti-inflammatory and liver protection. In this study, we explored whether SA regulates oxidative stress and pyroptosis through BRD4 to play a protective effect in ALD. Male C57BL/6 mice and AML-12 cells were used for experiments. We found that SA treatment largely abolished the up-regulation of BRD4 and key proteins of the canonical pyroptosis signalling in the liver of mice fed with alcohol, while conversely enhanced the antioxidant response. Consistantly, both SA pretreatment and BRD4 knockdown inhibited oxidative stress, pyroptosis, and liver cell damage *in vitro*. More importantly, the expression levels of BRD4 and pyroptosis indicators increased significantly in ALD patients. Molecule docking analysis revealed a potent binding of SA with BRD4. In conclusion, this study demonstrates that SA reduces ALD through BRD4, which is a valuable lead compound that prevents the ALD process.

## Introduction

Long-term and excessive alcohol consumption can lead to progressive steatohepatitis and fibrosis, which further develops into liver cirrhosis and hepatocellular carcinoma. Among men, the proportion of global deaths caused by alcohol is 7.6%, among women it is 4.0%, and 139 million disability-adjusted life-years are lost due to drinking, accounting for 5.1% of the total global disease burden (Seitz et al., 2018; Singal et al., 2018). Alcohol-induced liver steatosis is initially manifested as the accumulation of small or large droplets in hepatocytes (Gao and Bataller, 2011). When alcohol continues to be overloaded, it will further aggravate liver damage, leading to inflammation, oxidative stress, and multiple pathways of programmed cell death (Rangwala et al., 2011; Ikura and Caldwell, 2015; Ma et al., 2020). Due to the complicated pathogenesis of alcohol-associated liver disease (ALD) and unclear underlying mechanism, is still there is no targeted treatment. Therefore, it is necessary to better understand the pathogenesis of ALD and develop new and safe drugs for the treatment.

Sinapic acid (SA, 4-hydroxy-3,5-dimethoxy cinnamic acid), one of the phenolic acid compounds, is the effective ingredients of traditional Chinese medicine. It is widely found in various oil crops, grains, vegetables, and berries. SA has anti-inflammatory, antioxidant, and antibacterial effects (Chen, 2016; Huang et al., 2018; Lee, 2018; Huang et al., 2020). It has been proved that SA has a strong effect on reducing the oxidative stress of the liver and colon of rats fed high-fat diet (Yang et al., 2019) and abating the pyroptosis in diabetic atherosclerosis (Han et al., 2018). Shin et al. demonstrated that SA protected the rat liver from CCl4-induced inflammation and exhibited antifibrotic effects against DMN-induced liver injury (Shin et al., 2013b; Shin et al., 2013a). However, the efficacy of SA in prevention of alcohol-associated liver disease has not yet been studied.

Oxidative stress has been identified as a potential mechanism of ALD, which is caused by the imbalance between antioxidant capacity and reactive oxygen species (ROS) (Hsu et al., 2020). The nuclear factor-erythroid 2-related factor (Nrf2) is considered to be a cytoprotective factor, and to a certain extent it exerts anti-oxidant and anti-inflammatory effects through heme oxygenase-1 (HO-1) and their products (Loboda et al., 2016). In addition to oxidative stress, multiple forms of cell death also play the considerable role in the complicated ALD pathogenesis. Recently, pyroptosis, a new type of inflammatory programmed cell death was discovered in ALD, which also provides new ideas for the treatment of ALD (Heo et al., 2019; Kai et al., 2020).

Bromodomain-containing protein 4 (BRD4) is the most widely studied member of the bromodomain and extra terminal domain-containing (BET) protein family. Recently, we first determined that BRD4-induced inflammatory response is a split-new access for ALD (Lan et al., 2020). Studies have shown that BRD4 can be regarded as a therapeutic target in many diseases related to oxidative stress and pyroptosis, such as renal ischemia/reperfusion injury, macrophage dysfunction, acute gouty arthritis and renal cell carcinoma (Liu et al., 2019a; Hao et al., 2020; Tan et al., 2020; Zhu et al., 2020). However, the potential role of BRD4 in ALD needs to be further clarified.

In this study, we first explored whether SA can protect against ALD. Subsequently, molecular docking and biochemical evaluation were applied to verify the binding mode of SA and BRD4. It is revealed that SA targets BRD4 to alleviate the oxidative stress and pyroptosis of ALD.

## Materials and Methods

### Animals and Treatments

Male C57BL/6 mice (8 weeks old) were obtained from the Animal Center of Dalian Medical University (Dalian, China). All animal procedures were performed in accordance with the Guidelines for the Care and Use of Laboratory Animals and were approved by the Institutional Ethics Committee of Dalian Medical University (Dalian, China). SA (>98% purity) and sodium carboxy methyl cellulose (CMC-Na) were separately purchased from Sigma-Aldrich (Missouri, United States ) and Solarbio (Beijing, China). SA is suspended in 0.1% CMC-Na solution before use. The mice were adapted for one week before the experiment and then fed either a normal diet (control) or a Lieber-DeCarli liquid diet containing 5% ethanol (v/v) (EtOH) for 8 weeks. Liquid diets were freshly prepared before distribution. Sixty mice were equally divided into five groups, including control group, control + SA (20 mg kg^−1^ per day) group, EtOH group, and two groups of SA-treated EtOH mice that were respectively administrated (i.g.) with SA at the dosages of 10 and 20 mg kg^−1^ per day. Subsequently, blood was gathered from the abdominal aorta and liver tissues were collected for further analysis.

### Human Liver Samples

Liver samples of human ALD patients were obtained from the Second Affiliated Hospital of Dalian Medical University (Dalian, China), and were handled under the approval of the Ethics Committee of Dalian Medical University. All patients signed informed consent forms. The basic biological information of controls and patients were presented in Supplementary Table S1.

### Cell Culture and Treatment

Mouse hepatocyte cell line AML-12 cells (American Type Culture Collection (ATCC), Manassas, VA, United States) were maintained at 37 °C in a humidified atmosphere with 5% CO_2_, and cultured in a 1:1 mixture of Dulbecco’s modified Eagle’s medium/Ham’s F-12 medium (Gibco, New York, United States) containing 40 ng/ml dexamethasone, 5 μg/ml Insulin-Transferrin-Selenium (Sigma-Aldrich, Missouri, United States), and 10% fetal bovine serum (Gibco, New York, United States).

Before protein extraction, cells were pretreated with 20 μM SA for 6 h, 100 mM ethanol, or neither (control) for 24 h. Small interfering RNA (siRNA)was designed and synthesized by GenePharma (Shanghai, China). Lipofectamine 3,000 (Invitrogen) was used for transfection. The sequences of the BRD4 siRNA were as follows: sense 5′- GCC​UGA​GAU​GAA​GCC​UGU​ATT -3′, antisense 5′- UAC​AGG​CUU​CAU​CUC​AGG​CTT -3′. After transfection for 24 h, the cells were incubated either with or without 20 μM SA for 6 h and either with or without ethanol for 24 h. Finally, the cells were harvested and processed for protein extraction.

### Cell Viability

Cell viability was quantitatively analyzed with the Cell Counting Kit-8 (CCK-8, Dojindo, Tokyo, Japan). The experiments were carried out in 96-well plates, incubated at 37 °C for 2 h, and then optical density (OD) values were measured with Thermo Multiskan FC microplate luminometerat 450 nm.

### Biochemical Analysis

Alanine aminotransferase (ALT), aspartate aminotransferase (AST), total cholesterol (TC), triglyceride (TG) and alcohol dehydrogenase (ADH) levels were assayed in serum, while malondialdehyde (MDA) and glutathione (GSH) levels were determined in liver tissues by commercial kits according to the manufacturer’s instructions (Nanjing Jiancheng Corp., Nanjing, China).

### Histological Analysis

Isolated liver lobes were fixed in 4% paraformaldehyde at room temperature at least overnight and then embedded in paraffin for hematoxylin-eosin (H&E) or used to prepare frozen sections for Oil Red O staining. The sections were examined by light microscopy.

For immunohistochemistry, antigen retrieval was performed in a Tris/EDTA buffer (pH 9). Then, the sections were incubated with F4/80, NLRP3 or caspase-1 primary antibody (Abcam, Cambridge, United Kingdom), followed by the corresponding secondary antibody. After visualizing with 3,3-diaminobenzidine (DAB) colour-rendering solution, the sections were counterstained with hematoxylin.

### Immunofluorescence Staining

Liver sections from ALD patients were incubated with anti-BRD4 primary antibody (Abcam, Cambridge, United Kingdom) overnight at 4 °C, followed by incubation with secondary antibody for 2 h at room temperature. Next, DAPI (Beyotime Institute of Biotechnology, Hangzhou, China) was used to stain nuclei.

### Western Blotting

The tissues and cells were lysed in Lysis Buffer for Western with 1 mM PMSF according to the manufacturer’s instructions (Beyotime, Shanghai, China). Total protein solution was separated by centrifuge at 4 °C for 10 min, and the supernatant was quantified with a BCA kit (Beyotime, Shanghai, China). Then, the supernatant was boiled for 10 min by adding loading buffer and separated by SDS-PAGE. The primary antibodies used for western blotting were as follows: BRD4, GSDMD, IL-18, Nrf2, HO-1 (all from Abcam, Cambridge, United Kingdom), caspase-1, IL-1β (both from Cell Signalling Technology, Boston, United States), NLRP3, SOD2, and *β*-actin (all from Proteintech, Wuhan, China).

### Molecular Docking Assay

The BRD4 protein structure (PDB: 3P5O) was taken from the Protein Data Bank (http://www.rcsb.org/pdb). The structure sequence was analyzed by PyMOL software to remove the original ligands and water molecules. Coordinates for both the ligand (SA) and target protein (BRD4) were prepared with AutoDock Tools-1.5.6, and molecular docking analysis of ligand-protein complexes was conducted using the AutoDock4.2. The docking results are presented after mapping with Discovery Studio version4.5.

### Nile Red Staining

To examine intracellular lipid accumulation, AML-12 cells were incubated with Nile red staining (Sigma, Missouri, United States). The images were observed with a fluorescence microscope.

#### DCFH-DA Staining

AML-12 cells were stained with 10 μM DCFH-DA staining (Sigma, Missouri, United States) for 30 min at 37 °C away from light. DCFH-DA fluorescence results were viewed using a fluorescence microscope.

### Statistical Analysis

The results are expressed as the mean ± SD. GraphPad Prism (GraphPad Prism Software, CA, United States) was used to analyze the data. Student’s unpaired *t*-test (two-group comparisons) and one-way ANOVA (multigroup comparisons) were executed. *p* < 0.05 was considered statistically significant.

## Results

### SA Attenuates Chronic Alcohol-Induced Liver Injury and Steatosis

**FIGURE 1:**
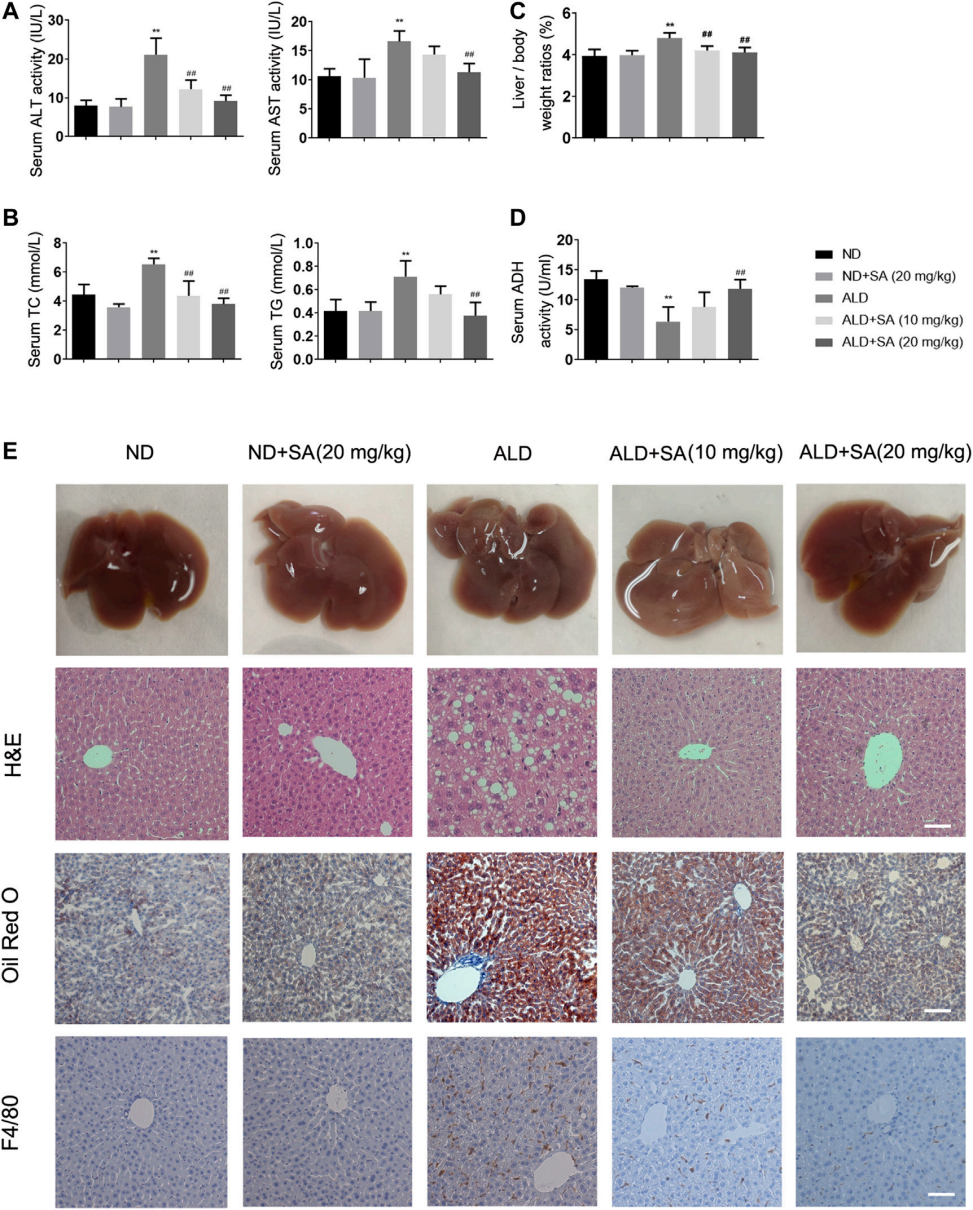
SA diminishes alcohol-induced liver injury and steatosis in mice. **(A)** Serum levels of ALT and AST. **(B)** Serum levels of TC and TG. **(C)** Liver/body weight ratio **(D)** Serum ADH levels. The data are the mean ± SD (*n* = 8). **(E)** Representative morphological, H&E staining (×200), Oil Red O staining (×200) and F4/80 staining (×200) of liver sections from the different experimental groups: normal diet (ND), ND + SA (20 mg/kg), Lieber-DeCarli diet (ALD), ALD + SA (10 mg/kg), and ALD + SA (20 mg/kg). Scale bar = 50 μm **p < 0.01 *vs* the ND group; ^##^ *p <* 0.01 *vs* the ALD group.

We first explored whether SA exerts a protective effect in alcohol-induced liver injury. Compared to the control, serum ALT, AST (Figure 1A), TC, TG (Figure 1B), the liver/body weight ratios (Figure 1C) were obviously increased after an ethanol diet, while serum ADH (Figure 1D) levels were decreased. SA treatment diminished the alcohol-induced liver injury in a dose-dependent manner. TNF-α mRNA levels, CYP2E1 protein expression levels and MCP-1 staining reflected the same fact (Supplementary Figures S1A–C). Further histology analysis by H&E staining and Oil Red O verified the effects of SA on protecting mice from alcohol-induced liver injury. The morphological and histological examination showed that the liver sizes were enlarged, the colour changed, lipid droplet accumulated in hepatocytes in the model group. SA, however, alleviated these changes and minimized the pathology. The mRNA levels of FASN, SREBP-1c, ACOX-1, and ADRP also confirmed the truth of SA regulating fatty acid metabolism (Supplementary Figure 1D). In addition, in immunostaining experiments, SA treatment reduced the F4/80 staining intensity of the liver of mice fed with alcohol (Figure 1E). These results indicate that SA effectively protects against alcohol-induced liver injury and steatosis in mice.

### SA Reduces Alcohol-Induced Liver Damage by Inhibiting Oxidative Stress

**FIGURE 2:**
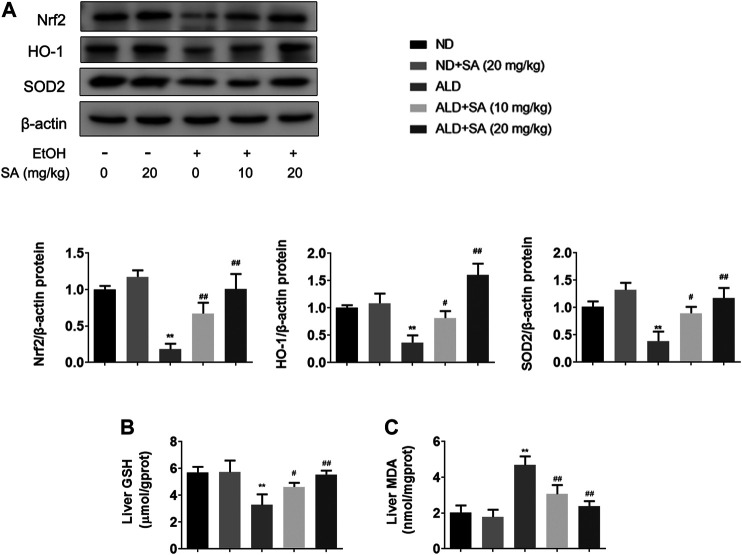
SA reduces alcohol-induced oxidative stress in mice livers. **(A)** Western blotting of hepatic Nrf2, HO-1 and SOD2 protein levels in livers (*n* = 3). **p < 0.01 *vs* the ND group; ^#^ *p <* 0.05, ^##^ *p <* 0.01 *vs* the ALD group. **(B)** Hepatic GSH levels (*n* = 6). **(C)** Hepatic MDA levels (*n* = 6). **p < 0.01 *vs* the ND group; ^#^ *p <* 0.05, ^##^ *p <* 0.01 *vs* the ALD group.

Oxidative stress is a main contributor to the pathogenesis of liver damage caused by alcohol. Indeed, mice that consumed alcohol showed decreased levels of antioxidant genes including Nrf2, HO-1, and SOD2 (Figure 2A) and hepatic GSH (Figure 2B) but represented high levels of hepatic MDA (Figure 2C). Furthermore, SA treatment significantly reversed the alcohol-mediated downregulation of protein expressions and GSH levels, whereas caused the decrease in MDA levels. Taken together, SA regulates alcohol-associated liver damage through suppressing oxidative stress related to Nrf2/HO-1 signalling.

### Alcohol-Induced Liver Damage Is Alleviated by SA Through Canonical Pyroptosis Signalling

**FIGURE 3:**
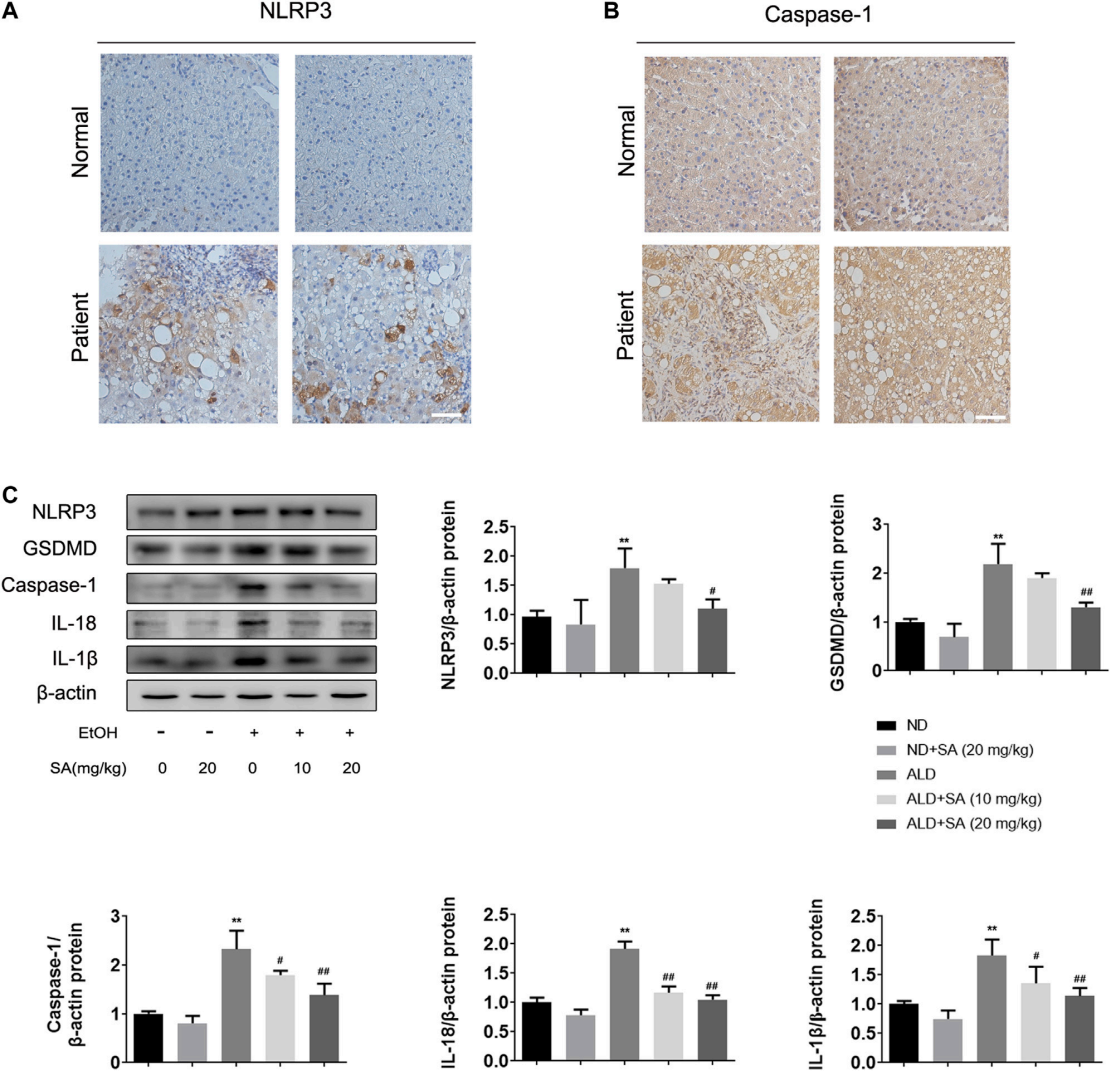
Alcohol-induced pyroptosis is alleviated by SA **(A)** IHC staining for NLRP3 inflammasome (×200) **(B)** IHC staining for caspase-1 (×200). Scale bar = 50 μm (*n* = 3) **(C)** Western blotting of hepatic NLRP3, GSDMD, activated caspase-1, IL-18 and IL-1β protein levels in livers (*n* = 3). ^**^ *p <* 0.01 *vs* the ND group; ^#^ *p <* 0.05, ^##^ *p <* 0.01 *vs* the ALD group.

In recent years, the key role of pyroptosis and inflammasome activation in the development of ALD has been shown by substantial evidence (Heo et al., 2019; Kai et al., 2020). Based on previous studies, the canonical pyroptosis signalling in ALD was detected. First, we examined NLRP3 inflammasome and caspase-1 expression by immunohistochemistry (IHC) staining in liver samples from ALD patients. In IHC, NLRP3 and caspase-1 staining intensities were increased in ALD patients (Figures 3A,B), which was further confirmed by western blot analysis in mice. The protein levels of key regulatory proteins in canonical cell pyroptosis, including NLRP3, activated caspase-1 and GSDMD were increased by ethanol and declined by SA in a dose-dependent manner. Western blot analyses also demonstrated that SA could reduce the levels of activated IL-18 and IL-1β in ALD mice, which were the substrate of caspase-1 and most commonly used pyroptosis inflammatory marker (Figure 3C). To sum up, the above data indicate that SA could attenuate ethanol induced pyroptosis via canonical caspase-1 pathway.

### The Protective Effect of SA in ALD Is Related to BRD4

**FIGURE 4:**
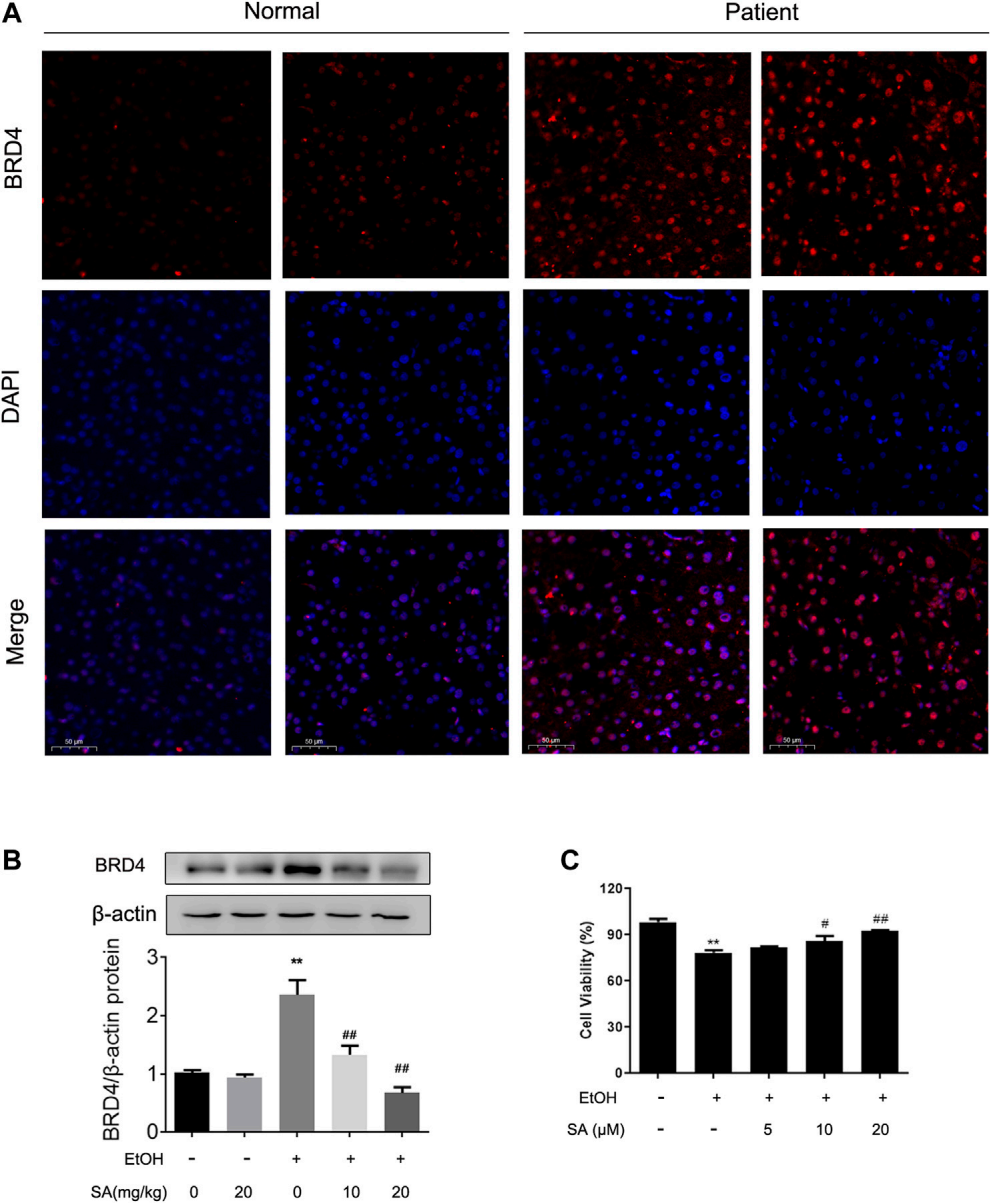
Effects of SA on BRD4 protein level *in vivo* and *in vitro* **(A)** Immunofluorescence analysis of BRD4 protein expression in human (×200). Scale bar = 50 μm (*n* = 3). **(B)** Western blotting of hepatic BRD4 protein levels in mice (*n* = 3). **p < 0.01 *vs* the ND group; ^##^ *p <* 0.01 *vs* the ALD group. **(C)** AML-12 cells were pretreated with 5, 10, or 20 μM SA for 6 h before exposure to EtOH (100 mM) for another 24 h. The viable cells were determined by the CCK8 assay. **p < 0.01 *vs* the control group; ^#^ *p <* 0.05, ^##^ *p <* 0.01 *vs* the EtOH group.

Simultaneously, our previous studies have confirmed that BRD4 expression can be stimulated in ALD (Lan et al., 2020). In order to determine the biological role of BRD4 in alcohol-associated liver disease in clinical setting, BRD4 expression was compared assayed in liver biopsy samples between healthy controls and ALD patients by immunofluorescence. Consistent with previous research, BRD4 expression was increased in livers from ALD patients (Figure 4A). Western blotting also validated the increase of BRD4 expression levels in alcohol-exposed mice, and the dose-dependent decrease by SA treatment (Figure 4B)*.* Based on the above findings, AML-12 cells were induced by EtOH for 24 h to establish the ALD model *in vitro*. AML-12 cells transfected with BRD4 siRNA, and then different concentrations of SA pretreated the cells. *In vitro* we got the same trend, 20 μM SA was determined for the following experiments (Figure 4C). These results indicate that BRD4 is involved in the effect of SA on ALD.

### SA Prevents Alcohol-Induced Hepatocyte Injury and Steatosis Through BRD4

**FIGURE 5:**
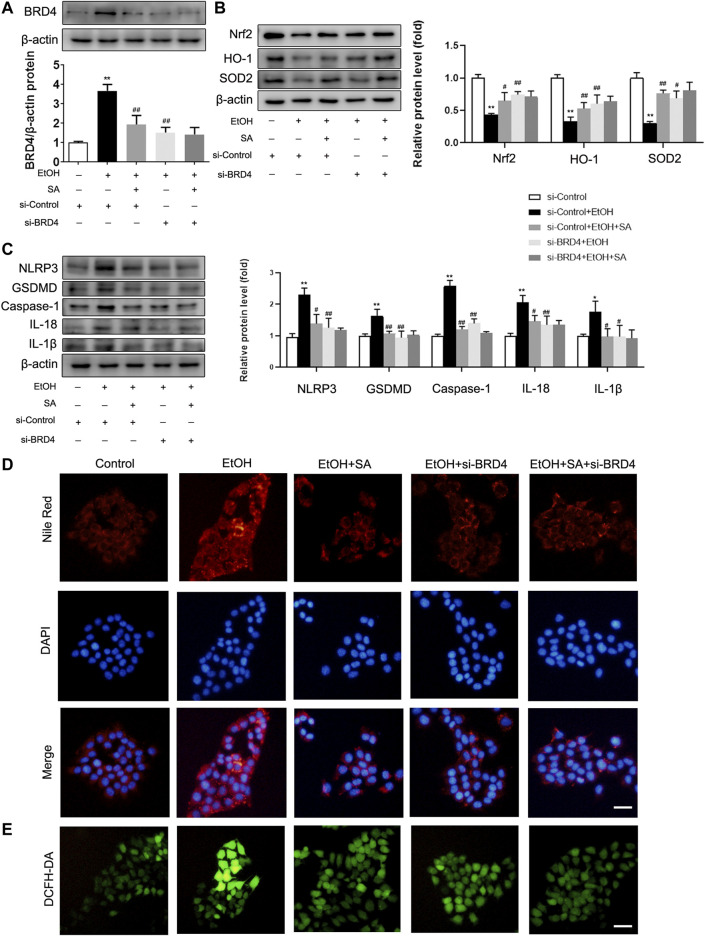
SA ameliorates oxidative stress, pyroptosis and reduces lipid accumulation in EtOH-treated AML-12 cells by regulating BRD4. **(A)** AML-12 cells were transfected with BRD4 siRNA or control siRNA for 24 h, 20 μM SA for 6 h, before exposure to EtOH (100 mM) for 24 h or not. Expression of BRD4 was measured *in vitro* (*n* = 3). **(B)** Western blotting of Nrf2, HO-1, and SOD2 levels (*n* = 3). **(C)** Western blotting of NLRP3, GSDMD, activated caspase-1, IL-18 and IL-1β protein levels (*n* = 3). *p < 0.05, **p < 0.01 *vs* the control group; ^#^ *p <* 0.05, ^##^ *p <* 0.01 *vs* the EtOH group. **(D)** Intracellular lipid accumulation was measured by Nile Red staining (×200). Scale bar = 50 μm. **(E)** DCFH-DA staining (×200). Scale bar = 50 μm.

In AML-12 cells, after EtOH treatment, the expression of BRD4 in the cells was significantly increased, SA treatment and si-BRD4 both down-regulated the increase (Figure 5A). Similarly, EtOH treatment resulted the decrease of Nrf2, HO-1 and SOD2, and SA treatment upregulated the expression of Nrf2, HO-1 and SOD2. BRD4 knockdown had a similar effect as that of SA treatment in suppressing the oxidative stress induced by ethanol (Figure 5B). At the same time, trends of the key regulatory proteins in pyroptosis, NLRP3, caspase-1, GSDMD, IL-18 and IL-1β, were consistent with that *in vivo* experiments. Moreover, BRD4 knockdown did not further reduce the pyroptosis related proteins in AML-12 cells treated with SA (Figure 5C). Meanwhile, BRD4 also played a similar role in HepG2 cells and Raw 264.7 cells (Supplementary Figures S2, 3).

The fluorescent stain results also confirmed our hypothesis. The number of lipid droplets in the EtOH group were significantly increased observed by Nile Red staining, SA treatment and BRD4 inhibition both reduced this increase (Figure 5D). DCFH-DA staining also indicated increased intracellular ROS in ALD. However, SA and BRD4 inhibition notably inhibited ROS formation in ALD (Figure 5E). Therefore, these findings indicate that BRD4 down-regulation mediates the protective effect of SA in alcohol-induced hepatocyte injury and steatosis.

### Specify Domain Interactions Between SA and BRD4

**FIGURE 6:**
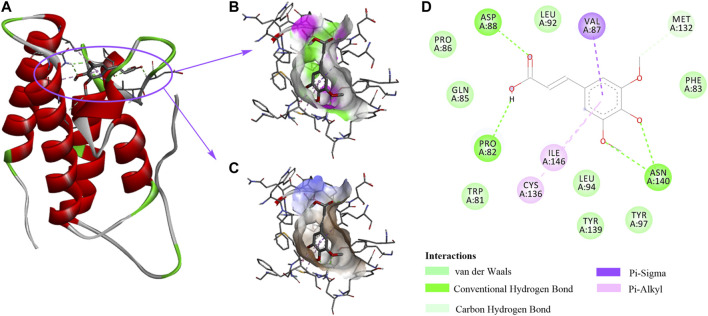
The interactions between SA and BRD4. **(A)** 3D structure of BRD4 (PDB:3P5O) in complex with SA. **(B)** The strong hydrophilic regions are green. **(C)** The strong hydrophobic regions are orange. **(D)** The BRD4 and SA interaction network is composed of van der Waals, Hydrogen bonds, Carbon Hydrogen bond, and π-ineractions.

Our data suggest that SA protects alcohol-induced liver damage through BRD4 suppression, implying that SA is a BRD4-specific inhibitor. We then applied molecular docking calculation technology to study the specific binding between SA and BRD4 (Figure 6A). The hydrophilic -OH groups in SA formed hydrogen bonds with Asp88, Pro82 and Asn140 (Figure 6B). In addition to hydrogen bonds, benzene ring can form hydrophobic interactions with amino acids residues Val87, Ile146 and Cys136 through π-σ and π-alkyl interactions (Figure 6C). SA contacts with nearby Met132 through carbon-hydrogen bonds, and contacts with Pro86, Gln85, Trp81, Leu94, Tyr139, Tyr97, and Phe83 through van der Waals interactions (Figure 6D).

Consistently, research showed that most BRD4 inhibitors imitate acetyl-lysine, occupy the central hydrophobic cavity, and form hydrogen bonds with Asn140 and Tyr97 directly or indirectly. Inhibitors can also extend to the ZA ring hydrophobic region, including the Trp/Pro/Phe motif, and the ZA channel, especially Pro82 to Leu91 in BRD4 BD1 through replacing benzene ring. To sum up, the binding ability of BRD4 and SA explains the mechanism by which SA relieves alcohol-associated liver disease.

## Discussion

The pathogenesis of ALD is complicated, involving ethanol metabolism, oxidative stress, gut microbiota imbalance, pyroptosis, lipid accumulation etc., which are widely considered to be important causes of clinical disease. Therefore, in-depth research on molecular mechanisms and effective drug treatments are necessary for the development of potential therapeutic methods. The current study presents the first attempt to demonstrate that SA contributes to the protection against ALD by reducing BRD4.

Natural compounds such as flavonoids, resveratrol, saponins, and *ß*-carotene have shown the protective effect on liver injury, through various mechanisms composed of antioxidative, anti-inflammatory, anti-apoptotic and so on (Tu et al., 2019). Our previous studies have verified that phenolic compound protocatechuic acid, salvianic acid A and salvianic acid B could reduce ALD (Zhang et al., 2017; Fu et al., 2019; Lan et al., 2020). SA is a phenolic compound commonly found in Chinese herbal medicine. In recent years, it has been reported that SA ameliorated liver disease and showed a protective effect on multiple organ diseases (Li et al., 2019; Aldubayan et al., 2020; Yun and Yang, 2020). However, whether SA treatment can relieve ALD is unclear. In this study, we used Lieber-DeCarli intake to establish an ALD model, and for the first time found that SA treatment can inhibit ALD indicated by the reduced levels of serum indicators and improved liver pathology. In addition, SA can prevent alcohol-associated liver disease by inhibiting pyroptosis and oxidative stress. Furthermore, the *in vitro* data also confirmed our above results. SA improved the survival rate of cells and prevented the progression of ALD. Therefore, we continued to explore the mechanism of SA to protect ALD.

Recent evidence shows that BRD4 not only plays a role in transcription regulation, but also has a non-transcriptional role in controlling DNA damage checkpoint activation and repair as well as telomere regulation (Donati et al., 2018). Inhibition of BRD4 can alleviate a variety of diseases, and our previous research has proved the vital role of BRD4 in chronic liver disease (Liu et al., 2019b; Hao et al., 2020; Lan et al., 2020; Tan et al., 2020; Zhu et al., 2020). Intriguingly, BRD4 could regulate the homeostasis of cardiomyocyte mitochondria and is essential for maintaining normal heart function (Kim et al., 2020). In this study, our data showed that lack of BRD4 remarkably reduced liver lipid droplets accumulation and ROS, which was similar to the results obtained after SA treatment. Therefore, we suggest that targeting BRD4 may represent a therapeutic approach for attenuating ethanol-induced liver damage. Due to strong hydrogen bonding, hydrophobic and van der Waals interactions, molecular docking analysis confirmed that SA exhibits a strong affinity for BRD4, suggesting that SA may be a promising activator by binding BRD4 in ALD.

Hepatocyte death is caused by apoptosis, necrosis, autophagy, necroptosis, pyroptosis, or the combination of its complex balance (Mazzolini et al., 2016). BRD4 positively regulates necroptosis through modulating the necroptosis executor mixed-lineage kinase domain-like (MLKL) expression and BET inhibitors have potential in the treatment of necroptosis-related diseases (Xiong et al., 2019). Pyroptosis leads to the formation of pores in the plasma membrane, cell swelling and the release of pro-inflammatory cell contents (Jorgensen and Miao, 2015). Hepatocyte pyroptosis and the inflammasome activation are also new mechanisms for the development of liver fibrosis, non-alcoholic steatohepatitis and ALD (Gaul et al., 2020; Mai et al., 2020). Moreover, Heo et al. has revealed ethanol promotes the overexpression of TXNIP and the activation of NLRP3 inflammasome, thereby inducing canonical cell pyroptosis (Heo et al., 2019), whereas Khanova et al. reported that non-canonical caspase-11/GSDMD pathway but not canonical caspase-1/IL-1β pathway is activated in alcoholic hepatitis (Khanova et al., 2018). Probable explanation may be due to the above-mentioned unclear boundary from chronic alcoholic steatohepatitis to alcoholic hepatitis and the differences in experimental conditions. In this study, our data showed that canonical pyroptosis pathway exist in our model, and the patient's immunohistochemical results are also in line with expectations. When BRD4 was inhibited, NLRP3/Caspase-1/GSDMD are all decreased, which is similar to the previous study: JQ1 inhibits BRD4 and reduces the LPS-induced acute colon injury by NLRP3/ASC/Caspase-1 inflammatory pyroptosis composition (Chen et al., 2021). Of interest, BRD4 might not participate in the activation of NLRP3 in bone marrow–derived macrophages (BMDMs), but it is an important regulator for the NLRC4 inflammasome activation in response to *S. typhimurium* infection. This is possibly due to BRD4 selectively activates a subset of NF-κB target genes (Dong et al., 2021). Nevertheless, the precise molecular mechanisms by which BRD4 promotes pyroptosis in ALD remain as an interesting open question, which is worthy of further investigation.

Oxidative stress is a process in which organisms or cells produce excessive ROS and then destroy proteins and DNA (S Narasimhan et al., 2020). Studies have shown that oxidative stress is inseparable from the pathogenesis of both acute and chronic ALD, and Nrf2/HO-1 signalling pathway takes part in oxidation resistance process. The liver protein expression of Nrf2 in ALD mice is significantly reduced (Wang et al., 2020). Similarly, ethanol administration can lead to decreased Nrf2 and HO-1 expression in livers from both chronic and acute model (Zeng et al., 2017). Interestingly, the liver expression of HO-1 in acute alcoholic liver damage may also increase in the short term (Liu et al., 2019a)*.* In our current research, BRD4 expression as determined by western blot was increased in ALD and was greatly reduced by antioxidant SA treatment. Here we also showed that BRD4 inhibited oxidative stress in ALD, and the level of anti-oxidative stress increased after knocking down BRD4. It has been shown that NADPH oxidase 4 (Nox4) is downregulated in idiopathic pulmonary fibrosis myofibroblasts by Brd4 inhibition, (Sanders et al., 2020). In addition, BRD4 could directly associates to chromatin regulatory regions of the NADPH oxidase subunits and BET inhibitors dramatically reduces oxidative stress and ameliorates skeletal muscle homeostasis and muscle function (Segatto et al., 2020). However, additional molecular pathways involved in BRD4 anti-oxidant functions cannot be excluded, which deserves further study.

In aggregate, our results demonstrated for the first time that SA could be a promising therapeutic agent for ALD via BRD4, which reduces oxidative stress and pyroptosis. This study may provide a new strategy for the treatment of alcohol-associated liver disease.

## Data Availability

The original contributions presented in the study are included in the article/Supplementary Material, further inquiries can be directed to the corresponding authors.
