# Supplementary material for: Sinapic Acid Reduces Oxidative Stress and Pyroptosis via Inhibition of BRD4 in Alcoholic Liver Disease
Source: Front Pharmacol. 2021 Jun 4;12:668708. doi: 10.3389/fphar.2021.668708 (PMC8212038; doi:10.3389/fphar.2021.668708)
Supplement: Supplementary file 1 [file Table1.DOCX]

**Supplementary Table 1. Clinical characteristics of patients.**

| **Parameter** | **Controls**  **(n = 7)** | **Alcoholic liver ­patients**  **(n = 6)** | **P-value** |
| --- | --- | --- | --- |
| Age (years) | 48.00 ± 5.725 | 46.82 ± 3.949 | P = 0.8632 |
| Sex (male/female) | 4/3 | 4/2 | - |
| ALT (U/L) | 38.88 ± 6.297 | 69.40 ± 20.02 | P < 0.01 |
| AST (U/L) | 34.70 ± 5.327 | 143.70 ± 26.43 | P < 0.01 |
| Creatinine (μmol/L) | 44.57 ± 6.430 | 61.27 ± 8.039 | P = 0.2584 |
| Total bilirubin (μmol/L) | 13.90 ± 1.554 | 29.78 ± 4.012 | P < 0.01 |
| Alkaline Phosphatase (U/L) | 76.54 ± 5.649 | 152.90 ± 13.47 | P < 0.01 |
| Albumin (g/L) | 46.92 ± 0.7713 | 37.27 ± 2.309 | P < 0.01 |
| Prothrombin Time (s) | 12.54 ± 0.2977 | 14.82 ± 0.8657 | P < 0.05 |
| Child-Pugh (A/B/C) | - | 0/3/3 | - |
