# Supplementary material for: Sinapic Acid Reduces Oxidative Stress and Pyroptosis via Inhibition of BRD4 in Alcoholic Liver Disease
Source: Front Pharmacol. 2021 Jun 4;12:668708. doi: 10.3389/fphar.2021.668708 (PMC8212038; doi:10.3389/fphar.2021.668708)
Supplement: Supplementary file 2 [file Table2.DOCX]

**Supplementary Table 2. Primer sequences.**

| **Gene** | **Forward primer** | **Reverse primer** |
| --- | --- | --- |
| **Human** | | |
| BRD4 | CTAAACTGGAGGCCCGTGAGT | CAAAGCGCATTTCGAACACA |
| SOD2 | TGGTGTCCAAGGCTCAGGTT | TAGTAAGCGTGCTCCCACACA |
| GSDMD | AGGAAGCCCTCAAGCTCATG | CCCTGTATCTGCCCATCCAT |
| β-actin | GGGAAATCGTGCGTGACATT | GGAACCGCTCATTGCCAAT |
| **Mouse** | | |
| BRD4 | CGATTGATGTTCTCCAACTGCTA | CTGCAGGAGAGGACACTGTAACAA |
| NLRP3 | CTGCGGACTGTCCCATCAAT | AGGTTGCAGAGCAGGTGCTT |
| GSDMD | CAGAACCAGAACCGGAGTGTTT | CATTCATGGAGGCACTGGAA |
| IL-6 | ACCACTCCCAACAGACCTGTCT | CAGATTGTTTTCTGCAAGTGCAT |
| TNF-α | ACAAGGCTGCCCCGACTAC | TGGGCTCATACCAGGGTTTG |
| FASN | CATGACCTCGTGATGAACGTGT | CGGGTGAGGACGTTTACAAA |
| SREBP-1c | TCAAAACCAGCCTCCCAAGA | CCCCGTCCACAAAGAAACG |
| ADRP | GTCTCGTGGGTGGAGTGGAA | GGCGTTGACCAGGACAGTCT |
| Acox-1 | ATGGTTTTCGTAAGGTCCTTCCT | CTGCGTCTGAAAATCCAAAATCT |
| β-actin | AGAGGGAAATCGTGCGTGAC | CAATAGTGATGACCTGGCCGT |
