# Supplementary material for: Sinapic Acid Reduces Oxidative Stress and Pyroptosis via Inhibition of BRD4 in Alcoholic Liver Disease
Source: Front Pharmacol. 2021 Jun 4;12:668708. doi: 10.3389/fphar.2021.668708 (PMC8212038; doi:10.3389/fphar.2021.668708)
Supplement: Supplementary file 3 [file DataSheet1.DOCX]

**Supplementary Materials**

**Western Blotting**

The primary antibodies used for CYP2E1 were from Proteintech, Wuhan, China.

**Histological Analysis**

For immunohistochemistry, the sections were incubated with MCP-1 primary antibody (Abcam, Cambridge, UK), followed by the corresponding secondary antibody.

**Real-time PCR**

RNAiso Plus (TaKaRa, Japan) was applied to isolate the total RNA. Then, cDNA synthesis and RNA amplification were carried out with a PrimeScriptTM RT reagent kit and SYBR Premix Ex TaqTM II (TaKaRa, Japan), respectively. Expression levels in each sample normalized to β-actin levels were determined by calculating ΔΔCt. Sequences of the primers used are shown in Supplementary Table 2.

**Cell Culture and Treatment**

The HepG2 human hepatoma cell line was cultured in MEM containing10% fetal bovine serum (Gibco, New York, USA). The cells were incubated at 37°C in humidified air with 5% CO_2_. After grown for 24 h, cells were pretreated with 20 μM SA for 6 h, or si-BRD4 for 24 h. Then, the cells were exposed to 100 mM ethanol for 48 h. Finally, the cells were harvested and processed for RNA extraction.

Si-BRD4 was transfected by Lipofectamine RNAi MAX (Invitrogen) into RAW264.7 cells (mouse macrophage cell line, American Type Culture Collection). Then the cells were treated with 20 μM SA for 6 h, exposed to ethanol (100 mM) for 24 hours prior to the treatment with LPS at 1000 ng/mL for 3 hours.
