# Supplementary material for: Sinapic Acid Reduces Oxidative Stress and Pyroptosis via Inhibition of BRD4 in Alcoholic Liver Disease
Source: Front Pharmacol. 2021 Jun 4;12:668708. doi: 10.3389/fphar.2021.668708 (PMC8212038; doi:10.3389/fphar.2021.668708)
Supplement: Supplementary file 4 [file Image1.pdf]

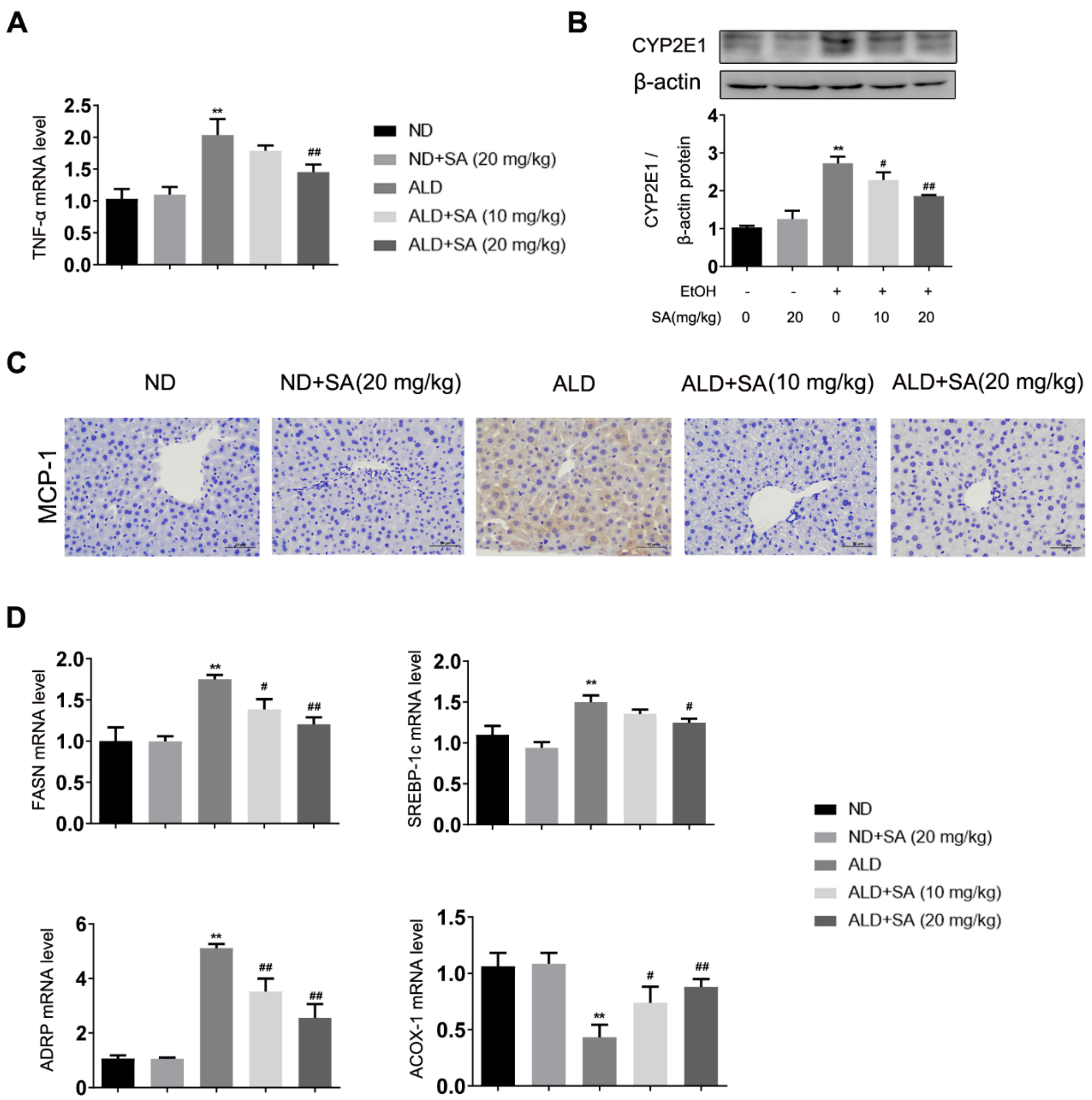

FIGURE S1. SA diminishes alcohol-induced liver injury in mice. **(A)** The mRNA levels of TNF-α in mouse livers (n = 6). **(B)** Western blotting of CYP2E1 levels (n = 3). **(C)** IHC staining for MCP-1 (×400). Scale bar = 50 μm. **(D)** The mRNA levels of FASN, SREBP-1c, ADRP and ACOX-1 in mouse livers (n = 6). ***P*<0.01 *vs* the ND group; $^{\#}$*P*<0.05, $^{\#\#}$*P*<0.01 *vs* the ALD group.

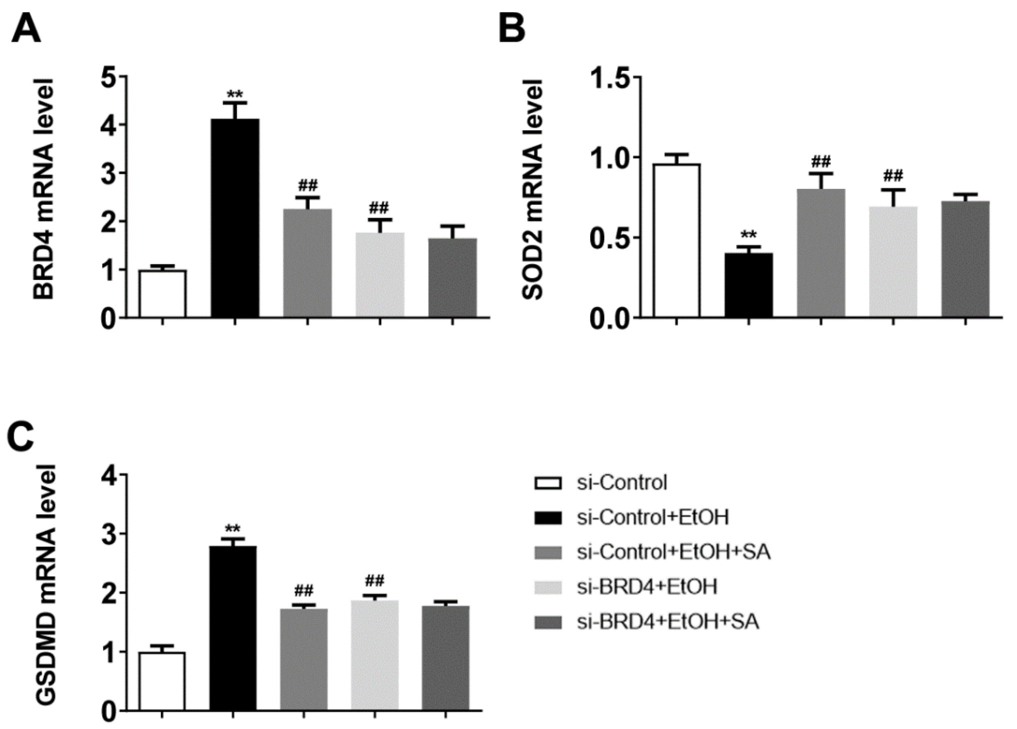

FIGURE S2. SA ameliorates oxidative stress and pyroptosis in EtOH-treated HepG2 cells. **(A)** The mRNA levels of BRD4 (n = 6). **(B)** The mRNA levels of SOD2 (n = 6). **(C)** The mRNA levels of GSDMD (n = 6). $^{**}P$<0.01 *vs* the control group; $^{\#\#}P$<0.01 *vs* the EtOH group.

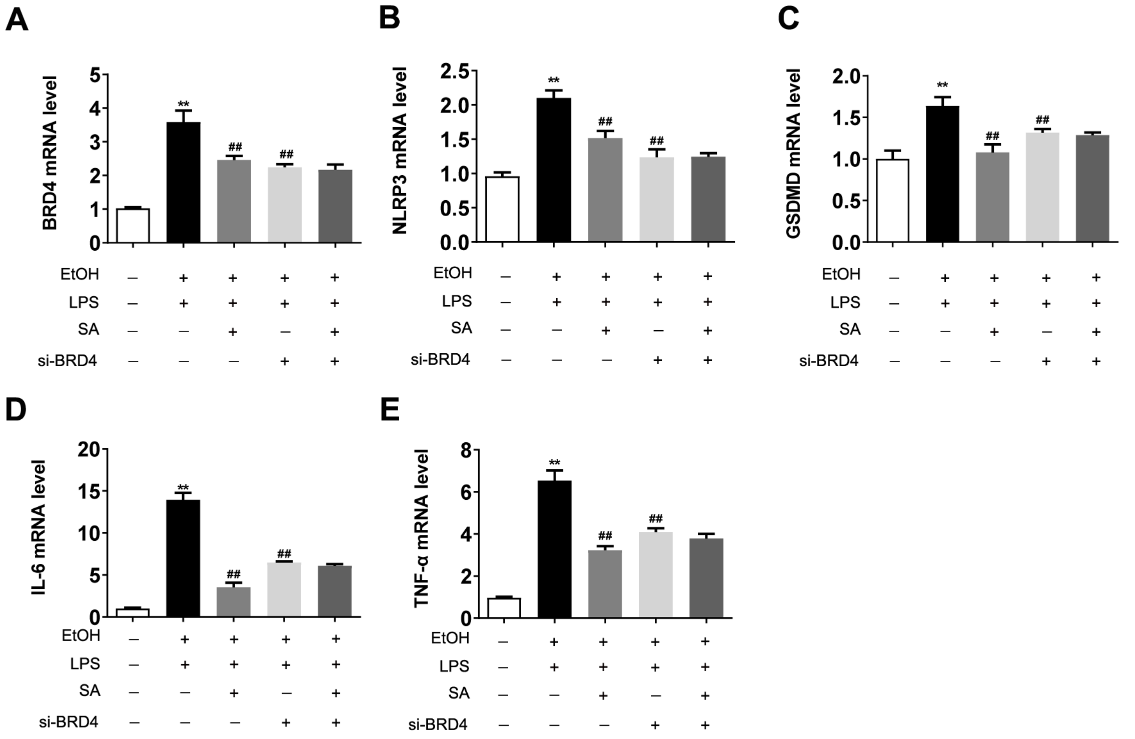

FIGURE S3. SA ameliorates pyroptosis and inflammation in Raw 264.7 cells by regulating BRD4. **(A)** The mRNA levels of BRD4 (n = 6). **(B)** The mRNA levels of NLRP3 (n = 6). **(C)** The mRNA levels of GSDMD (n = 6). **(D)** The mRNA levels of IL-6 (n = 6). **(E)** The mRNA levels of TNF-α (n = 6). $^{**}P<0.01$ *vs* the control group; $^{\#\#}P<0.01$ *vs* the EtOH + LPS group.
